# A comparative study of clinicopathological and imaging features of HBV-negative and HBV-positive intrahepatic cholangiocarcinoma patients with different pathologic differentiation degrees

**DOI:** 10.1038/s41598-023-47108-6

**Published:** 2023-11-13

**Authors:** Xiaoli Huang, Dan Yu, Xintao Gu, Jiansun Li, Jiaqi Chen, Yuanqiang Zou, Jinyuan Liao

**Affiliations:** 1 Department of Radiology, The First Affiliated Hospital of Guangxi Medical University, No. 6 Shuangyong Road, Nanning, 530021 Guangxi People’s Republic of China; 2 Department of Radiology, Yulin First People’s Hospital, No. 495 Jiaoyuzhong Road, Yulin, 537000, Guangxi China

**Keywords:** Liver cancer, Cancer imaging

## Abstract

Hepatitis B is a risk factor for the development of intrahepatic cholangiocarcinoma. The prognosis of HBV-related ICC remains to be further investigated. To investigate the clinical, pathological and imaging features of intrahepatic cholangiocarcinoma of hepatitis B virus-positive and -negative patients. Data from January 31, 2012 to December 31, 2019 of 138 patients were retrospectively analyzed. The patients were divided into hepatitis B virus-positive group (group A[n = 66]) and virus-negative group (group B[n = 72]), and the patients were divided into groups according to pathological differentiation degree and tumor size. The differences in clinical, imaging characteristics and the progression-free survival between groups were analyzed. There were significant differences in gender, age, HBc antibody, CA125 and AFP, tumor distribution site, maximum diameter, plain scan density, inferior hepatic angle, peritumoral bile duct dilatation, vascular encasement invasion, intrahepatic bile duct dilatation and lymphadenopathy between the two groups (*P* < 0.05); There were statistical differences in signs of vascular encasement invasion between the two groups with well-to-moderately differentiated tumors (*P* < 0.05); there were statistical differences in tumor density uniformity, signs of vascular encasement invasion and lymphadenopathy between the two groups with poorly differentiated tumors (*P* < 0.05). Large groups A and B showed differences in tumor density uniformity, vascular encasement invasion, arterial phase, overall reinforcement pattern, peritumoral bile duct stones and biliary dilatation (*P* < 0.05). There was no statistical difference in postoperative PFS between the two groups (*P* > 0.05). The clinical and imaging features of ICC of hepatitis B virus-positive and -negative patients are different, and there is little difference in postoperative disease-free survival time.

Intrahepatic cholangiocarcinoma (ICC) is the second most common primary hepatic malignancy after hepatocellular carcinoma (HCC) and originates from the secondary and above intrahepatic bile ducts due to malignant transformation of biliary epithelial cells^1,2^. Previous studies have investigated risk factors associated with intrahepatic cholangiocarcinoma, such as intrahepatic bile duct stones and liver fluke related infection^3–6^. In addition, hepatitis B is also a risk factor for the development of ICC^7–10^. The mechanism of hepatitis B virus-induced ICC may be that hepatitis B virus persistent infection on the liver, continuous stimulation of inflammation makes intrahepatic cholangiocytes malignant transformation to form tumors^11^; followed by hepatitis B virus hepatotropic, not easy to infect cholangiocytes, some ICCs are formed by malignant transformation of hepatic stem cells^12,13^.

Zhou and Lee et al. found that some clinical and imaging features of hepatitis B-related ICC and hepatitis B-related HCC were similar, such as an earlier age of onset, lesions mostly located in the right lobe of the liver on imaging, and mass type was more common on pathology, while they were quite different from the findings of non-hepatitis B ICC^14,15^. Serum AFP levels were higher in HBV-related ICC patients than in HBV-negative ICC^16^. Jeong et al.^17^ also found that patients with HBV-related ICC had a higher degree of cirrhosis, incidence of tumor capsule, and microvascular invasion rate, and their tumors were also poorly differentiated. The surgical principle of HBV-related ICC is similar to that of HBV-related HCC, but because ICC can spread and metastasize intrahepatic and extrahepatic along Glisson sheath and lymphatic vessels, the tumor boundary is unclear, which is more likely to lead to positive resection margins. Some scholars have proposed that HBV-related ICC has less lymph node metastasis rate and better prognosis, and routine lymph node dissection is not recommended^18^. Antiviral therapy (AVT) for HBV-related ICC is also very important. For patients with high viral load, AVT should be given to improve the prognosis of patients at the same time of surgical treatment. However, the impact of hepatitis B virus infection on the prognosis of ICC patients remains controversial. Zhang et al.^19^. showed that patients with HBV-related ICC had a better prognosis than HBV-negative ICC patients, and he concluded that HBV infection may protect ICC patients after activating the immune response. In addition, the better prognosis of HBV-related ICC may also be related to the regular follow-up of HBV patients, so that ICC can be detected and treated early. However, some researchers believe that HBV infection has no correlation^20^ or negative correlation^21^ with the prognosis of ICC. Therefore, the prognosis of HBV-related ICC remains to be further investigated, and the analysis of pathological imaging features of ICC in chronic hepatitis B infection is helpful for clinical decision.

2019 The World Health Organization (WHO) divided ICC into small bile duct type and bold cast type. There are differences in the clinical manifestations of ICC among the above pathological subtypes, but there are not many reports in the literature on the differences between ICC imaging features with or without chronic hepatitis B infection and its pathological differentiation, and postoperative progression-free survival. In this study, ICC was divided into groups according to the presence or absence of chronic hepatitis B infection and different pathological differentiation to investigate the clinical, pathological, imaging features and prognosis of chronic hepatitis B-related intrahepatic cholangiocarcinoma and provide help for accurate clinical diagnosis and treatment.

## Materials and methods

### Patients

This retrospective study was approved by the First Affiliated Hospital of Guangxi Medical University(Ethics approval No. 2022-E431-01)ethics committees, and patient informed consent was waived. From January 1, 2012 to December 31, 2019, the clinical, imaging and pathological data of patients with intrahepatic cholangiocarcinoma who underwent CT examination and hepatectomy in our hospital were retrospectively analyzed.

**Figure 1:**
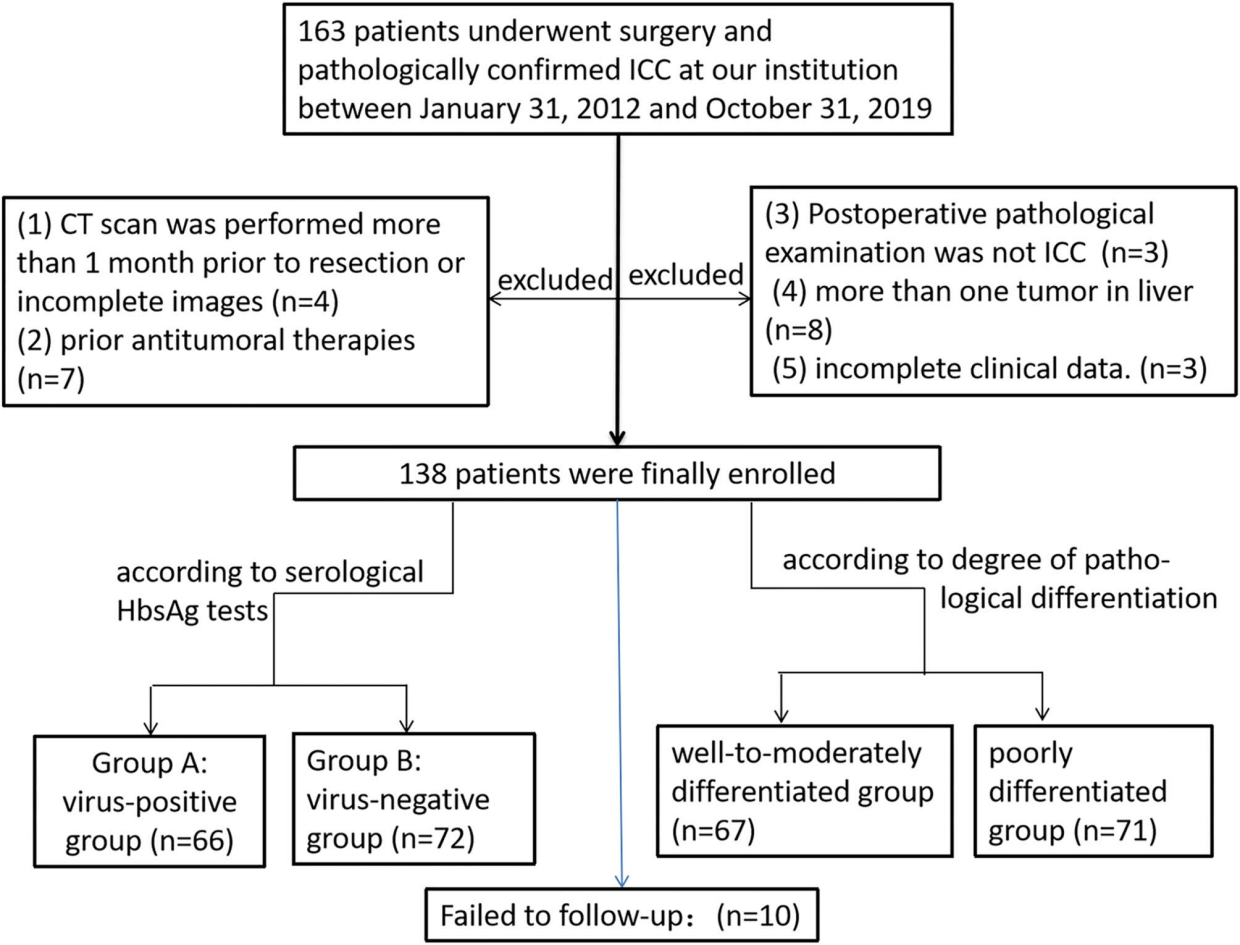
The flowchart shows the study group inclusion process. Numbers in parentheses are numbers of patients. ICC: intrahepatic cholangiocarcinoma, CT: computed tomography.

The main inclusion criteria: (1) All patients underwent radical resection without any prior antitumoral therapies; (2) CT scan was finished within 1 month prior to resection, including plain scan, arterial phase, portal venous phase and equilibrium phase images;(3) Postoperative pathological examination was ICC; (4) a single tumor in liver; (5) complete clinical data. Figure 1 summarizes the flowchart of the research work.

According to American Association for the study of liver diseeases(AASLD) 2018 guidelines for chronic hepatitis B^23^ , patients were divided into positive group (group A) and negative group (group B) according to whether they had chronic hepatitis B before treatment.

### CT scan

Simens dual-source spiral CT (SOMATOM Definition FLASH, Siemens Healthcare, Forchheim, Germany) was used. All patients underwent CT plain scan and enhanced scan. Before the examination, the patient fasted for 2 h. The scan parameters were as follows: tube voltage 120 kV, tube current 280 mA, rotation time 0.5 s, pitch factor 1.375. The scan was performed supine and ranged from the level of the right diaphragmatic dome to the level of the lower edge of the hepatosplenic region. Contrast-enhanced scanning: nonionic iodine solution was injected as the contrast medium with a high-pressure syringe through the cubital vein at a strength of 300 mg/ml, a dose of 1.5 ml/kg, and a administration rate of 3 ml/s. After the plain scans , three-phase contrast-enhanced scans of the liver were finished, including arterial phase (30 s), portal venous phase (60 s), and equilibrium phase (120 s).

### Image feature interpretation

All CT images were independently evaluated by two radiologists (with 5 and 6 years of clinical experience in abdominal MR imaging, respectively), who were blinded with regard to the clinical and histopathological information. In case of any discrepancy, a third abdominal radiologist (with 15 years of experience in abdominal diagnosis) was recruited to resolve.

**Figure 2:**
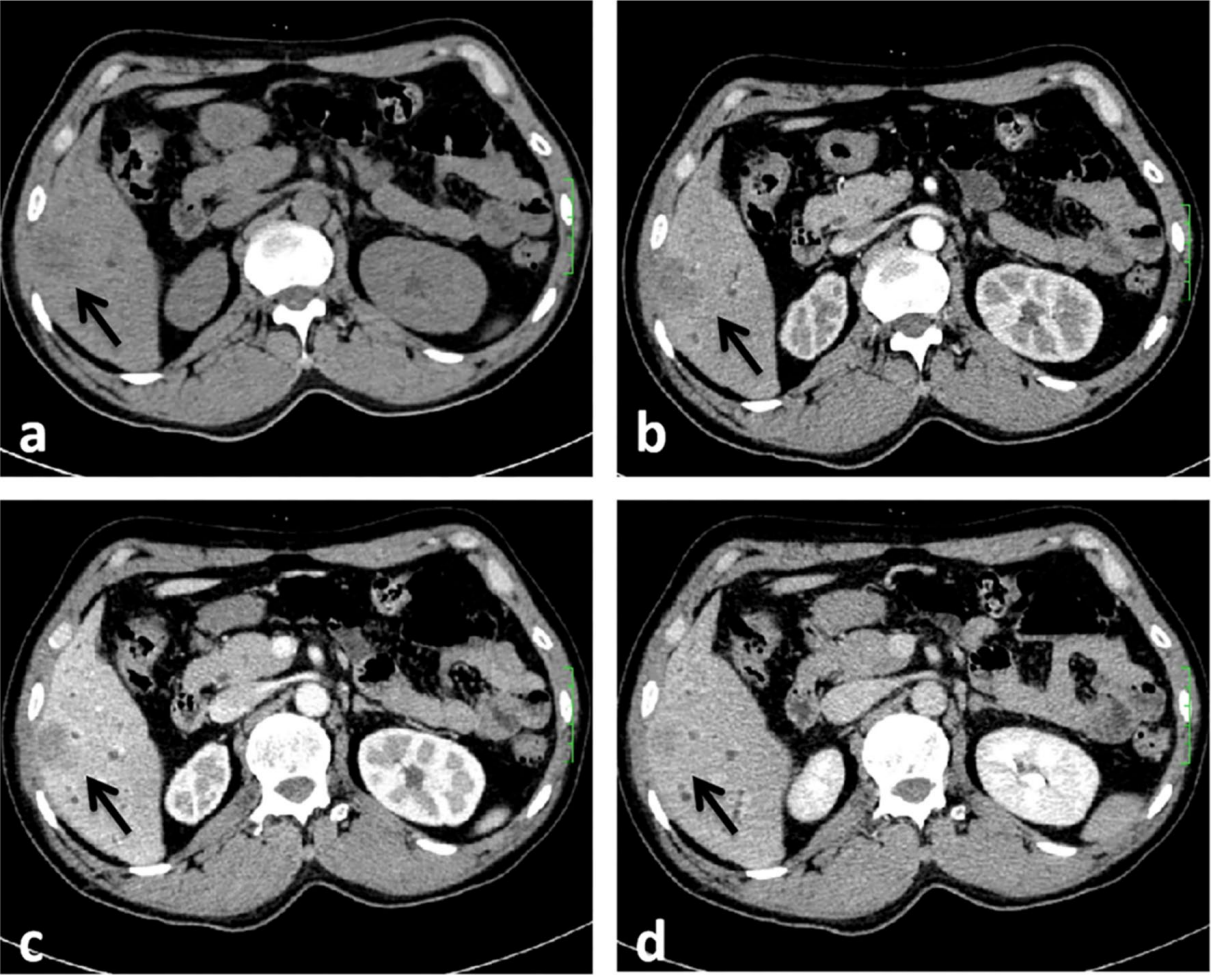
CT images in a 54-year-old HBV-negetive man with ICC confirmed by surgical resection, AFP 4.70 ng/ml CA19-9 14.09 U/ml, and its enhancement pattern was categorised as type I. Precontrast image (**a**) showed a solitary tumor (arrow) in segment VI of the liver; arterial phase (**b**) and portal phase (**c**) showed rim hyperenhancement; delayed phase (**d**) showed internal delayed hypoenhancement.

**Figure 3:**
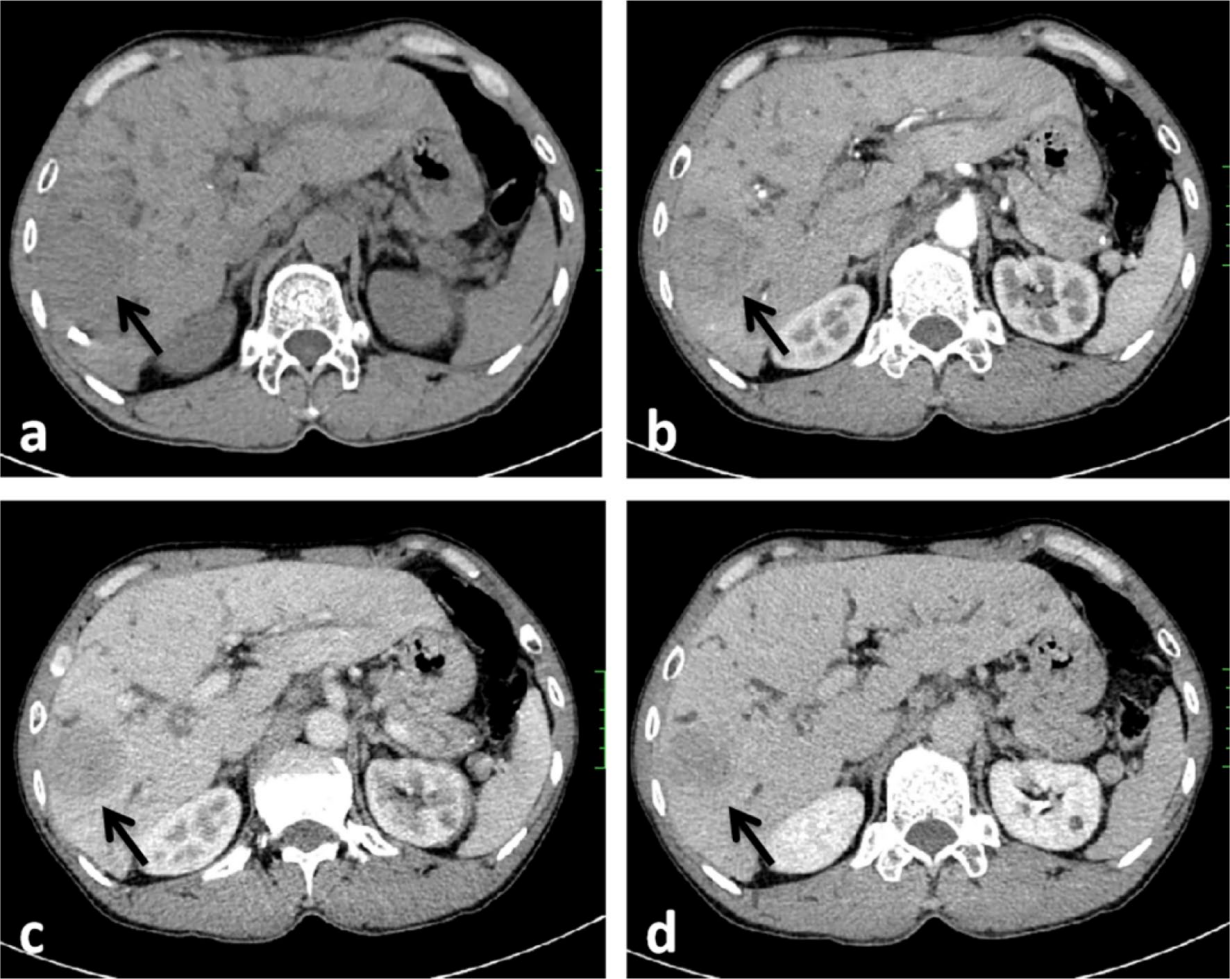
CT images in a 54-year-old HBV-negetive man with ICC confirmed by surgical resection, positive for schistosoma hepatica, AFP 6.56 ng/ml, CA19-9 < 2.00 U/ml, and its enhancement pattern was categorised as type II. Precontrast image (**a**) showed a solitary tumor (arrow) in segment VI of the liver; arterial phase (**b**), portal phase (**c**) and delayed phase (**d**) showed progressive hypoenhancement; portal phase (**c**) showed dilated intrahepatic bile ducts.

**Figure 4:**
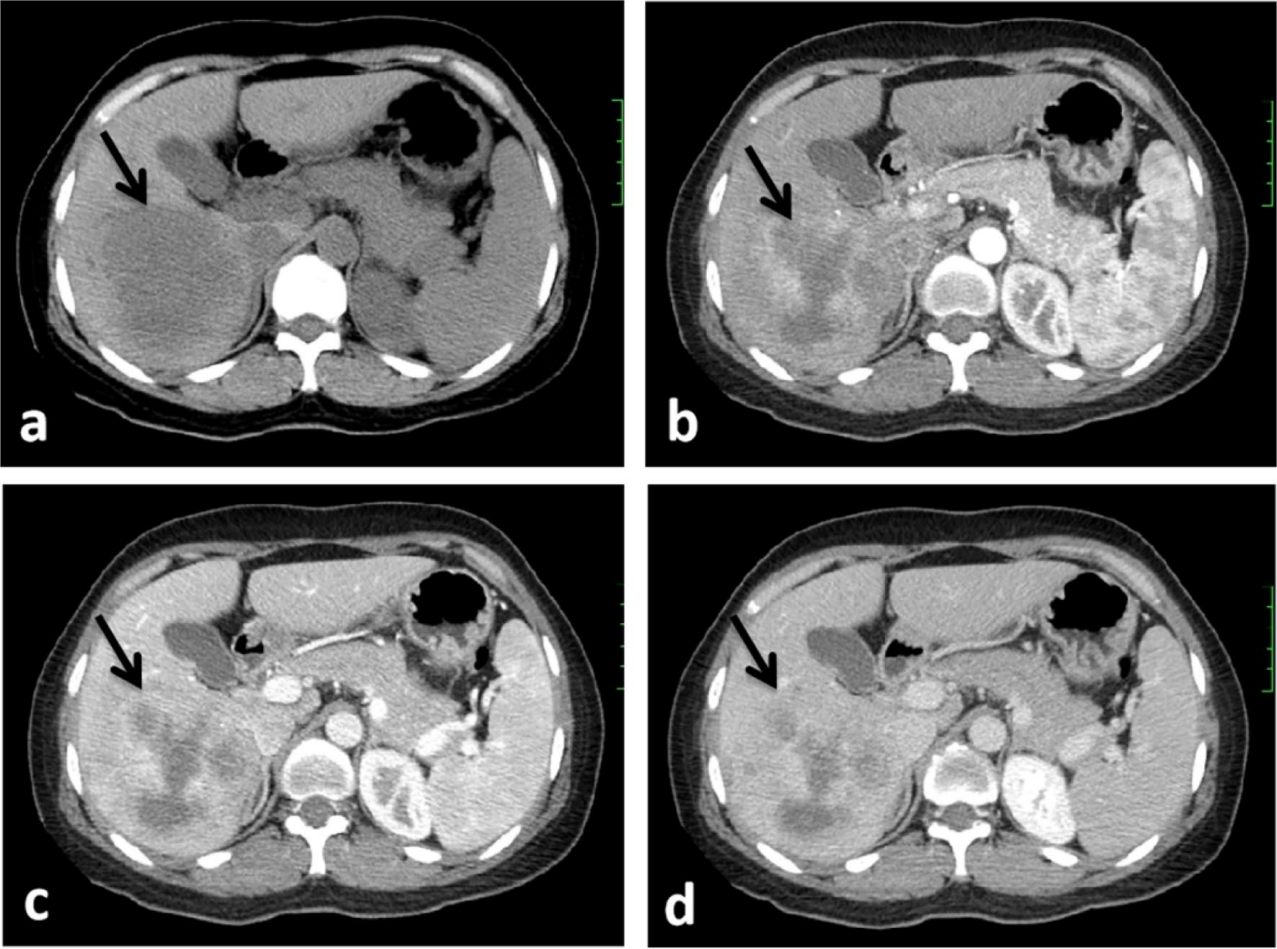
CT images in a 34-year-old HBV-positive woman with ICC confirmed by surgical resection, AFP 9.98 ng/ml, CA19-9 507.13U/ml, and its enhancement pattern was categorised as type III. Precontrast image (**a**) showed a solitary tumor (arrow) in the right lobe of the liver; arterial phase (**b**), portal phase (**c**) and delayed phase (**d**) showed rim hyperenhancement and central most necrosis.

**Figure 5:**
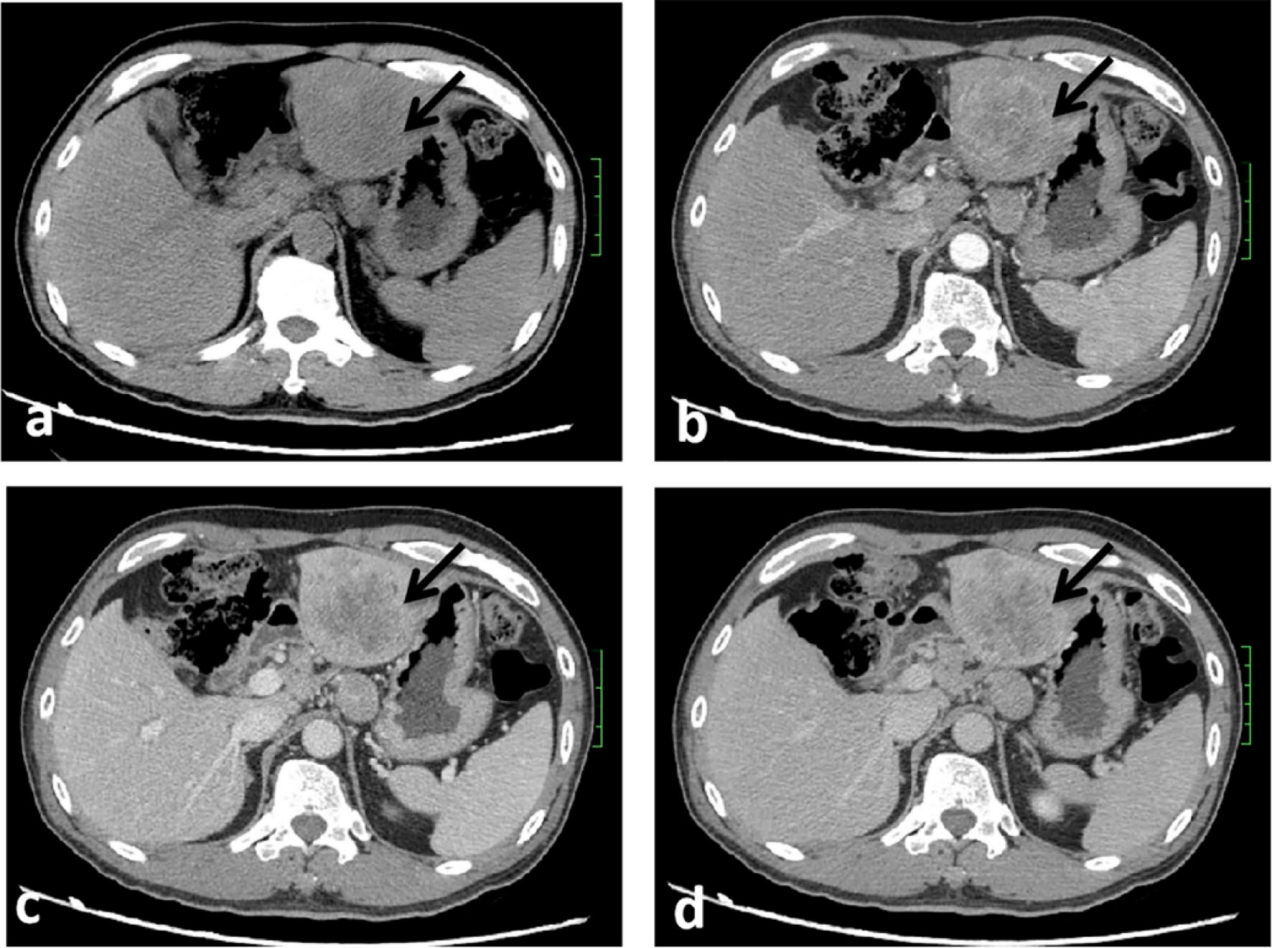
CT images in a 48-year-old HBV-positive man with ICC confirmed by surgical resection, AFP 3.79 ng/ml, CA19-9 163.6 U/ml, and its enhancement pattern was categorised as type IV. Precontrast image (**a**) showed a solitary tumor (arrow) in the left lobe of the liver; arterial phase (**b**) showed heterogeneous hyperenhancement; portal phase (**c**) and delayed phase (**d**) showed hypoenhancement.

The following imaging features, were evaluated: (1) Assessment of liver background: liver contour, sharpness of the lower edge of the liver, and esophageal-gastric varices; (2) General characteristics of the tumor: location, shape, size, boundary, and internal density; (Note: tumor size — the maximum diameter of the tumor was measured from the axial, coronal and sagittal views; the density was heterogeneous—there were cystic changes, necrosis, or calcifications in the lesion, and its volume exceeded 1/4 of the mass.) (3) Enhancement characteristics: According to previous study^24–26^ the enhancement pattern was divided into four types: arterial phase rim hyperenhancement + internal delayed hypoenhancement (type I), progressive hypoenhancement (type II), arterial phase rim hyperenhancement + central most necrosis (type III), and arterial phase heterogeneous hyperenhancement + portal or delayed phase hypoenhancement (type IV); (Note:the CT value of the lesion was compared with that of adjacent liver tissues, and the measurement range included more than 50% of the solid tumor area. When the difference between the two groups was less than -10 HU, it was defined as low density, when the difference between -10 HU and 10 HU, it was defined as isodense, and when the difference between the two groups was more than 10 HU, it was defined as high density; the overall enhancement degree decreased to arterial phase and portal venous phase or equilibrium phase CT value difference > 10 HU according to the comparison of their own CT value before and after tumor enhancement and the comparison of tumor CT value and liver parenchyma CT value. (4) Accompanying signs: peritumoral bile duct stones, peritumoral bile duct dilatation, shrinkage of the hepatic capsule, overall intrahepatic bile duct dilatation, common bile duct or left and right hepatic duct stones, gallstones or absence of the gallbladder, venous tumor thrombus and venous encasement by the tumor (veins refer to the inferior vena cava, portal vein, hepatic vein and its branches); (5) Abdominal lymph node assessment: range (subphrenic lymph node, hilar lymph node, gastrohepatic lymph node, periduodenal lymph node, peripancreatic lymph node, and para-aortic superior lymph node), size (short diameter > 1.0 cm is enlarged), homogeneity of enhancement (Non-homogeneous enhancement including heterogeneous enhancement and ring enhancement). Some representative cases are shown in Figs. 2, 3, 4 and 5.

### Follow-up after surgery

All patients were regularly followed up every 3 months in the outpatient department from discharge until the first occurrence of disease progression or death, whichever came first; the follow-up period ended in December 31, 2022.

### Clinical and pathological data evaluation

The analyzed clinical data comprised: (1) General data: gender, age, history of alcohol consumption and history of diabetes; (2) Main clinical symptoms and signs: abdominal pain, abdominal distension, fatigue, anorexia and jaundice.

The analyzed Laboratory parameters comprised: (1) Hepatitis B virological examination: hepatitis B surface antigen (HBsAg), hepatitis B surface antibody (HBsAb) and hepatitis B core antibody (HBcAb); (2) liver fluke infection examination (stool to find liver fluke eggs); (3) Gastrointestinal tumour markers: CEA, CA19-9, CA125, CA153 and AFP, PIVKA-II.

All tumor specimens were obtained by surgical resection and ICC was confirmed by gross microscopic histology, cytological morphology, and immunohistochemistry. According to the fourth edition of the WHO Classification of Tumours of the Digestive System, tumors were divided into well-to-moderately differentiated group and poorly differentiated group (Table 1). According to tumor maximum diameter, tumors were divided into small-to-medium group and large group.

**Table 1:** Group A and group B ICC pathological differentiation degree.

Pathological differentiation	Group A (n = 66)	Group B (n = 72)	P value
Well-to-moderately differentiated	27 (40.9%)	40 (55.6%)	0.085
Poorly differentiated	39	32	

Chi-square test

*Means *P*value is statistically different.

### Statistical analysis

All data were analyzed using SPSS 22.0 statistical software. Enumeration data were compared by Chi-square test or Fisher exact test, measurement data were compared by paired t-test or multivariate analysis of variance, and statistical results were analyzed by two-sided test; Kaplan–Meier survival curve was used to evaluate the relationship between postoperative progression-free survival in groups A and B, and Log Rank test was used for comparison between groups; when P < 0.05, the difference was statistically significant.

### Ethical approval

Research involves human participants. All data was from routine clinical test, and there was no clinical intervention for the participants in the study. This is a retrospective study in accordance with the Declaration of Helsinki and was approved by the Institutional Review Board of the First Affiliated Hospital of Guangxi Medical (Approve Number: 2022-E431-01) that waived the requirement of an informed consent.

### Informed consent

All informed consent for the routine clinical tests were obtained from participants. While for the retrospective nature of this study, informed consents for participating in this study was waived by the Institutional Review Board of the First Affiliated Hospital of Guangxi Medical.

## Results

### Clinicopathological characteristics

There were 66 patients in group A, including 43 males and 23 females, with an average age of 51.91 ± 8.86 years; 72 patients in group B, including 34 males and 38 females, with an average age of 55.42 ± 11.01 years; the differences in gender and age between the two groups were statistically significant (P = 0.040, 0.042). The odds ratio for gender being associated with chronic hepatitis B positivity was 2.432 (95% CI: 1.113–5.315).

The proportions of HBc antibody positive in the two groups were 100% and 78%, and the difference in HBc antibody was statistically significant (*P* < 0.05). (2) There was no statistically significant difference in the number of positive and negative cases of liver fluke infection (*P* > 0.05). (3) There was statistical difference in serum CA125 and AFP (*P* < 0.05), but there was no statistical difference in CEA, CA19-9 and CA153, PIVKA-II examination results (*P* > 0.05).

The gross pathology of the 138 cases, the texture was hard, the tumor section was mainly gr yelloayishw or grayish white, some scattered bleeding spots were observed, and the boundary of the lesion was mainly infiltrative. There were 67 cases of well-to-moderate differentiation, including 27 cases in group A and 40 cases in group B, and 71 cases of low differentiation, including 39 cases in group A and 32 cases in group B.

### Comparison of imaging between groups A and B

The imaging characteristics of ICC lesions in group A and group B are summarized in Table 2. Group A was mainly distributed in the right lobe and periphery of the liver, and group B was distributed in the left lobe and right lobe, periphery and near the hilum; the lesions in both groups were mainly round in shape, and blurred in border; the lesions were less than 3 cm in diameter in 7 patients (10.6%) and 1 patient (0.01%) in both groups, respectively. There were significant differences in tumor distribution sites and maximum lesion diameters between the two groups (*P* < 0.05); there was no significant difference in tumor shape and boundary (*P* > 0.05).

**Table 2:** Imaging characteristics of ICC lesions in group A and group B.

Observed items	Group A (n = 66)	Group B (n = 72)	*P* value
Tumor site			
Left lobe	13	34	0.003*
Right lobe	43	30	
Left + right lobe	10	8	
Tumor location			
Peripheral type	52 (78.8%)	42 (58.3%)	0.010*
Juxtahilar type	14	30	
Morpho-			
Round shape	44	43	0.399
Irregular shape	22	29	
Maximum diameter			
< 3 cm	7	1	0.039 *
3–6 cm	25	24	
> 6 cm	34	47	
Boundary			
Clear	33	32	0.609
Blurring	33	40	
Precontrast			
Non-uniform density	43	65	0.000*
Uniform density	23	7	
Arterial phase			
Edge ring high density	38	42	0.143
Non-uniform high density	10	4	
Equal/low density	18	26	
Overall enhancement pattern			
Type I	31	38	0.157
Type II	18	26	
Type III	7	4	
Type IV	10 (15.2%)	4 (0.05%)	

Chi-square test or Fisher 's exact test

*Represents that *P* values are statistically different.

In both groups, heterogeneous density was predominant on CT scans, slightly lower than peritumoral liver parenchymal density, and there was no statistically significant difference between the two groups (*P* > 0.05). Lesions in both groups showed predominantly circumferential hyperdensity or isodense hypodensity at the edges in the arterial phase, which was not statistically different (*P* > 0.05). There was no significant difference in overall enhancement pattern (*P* > 0.05). Group A showed 31, 18, 7 and 10 cases of type I–IV , respectively, and group B showed 38, 26, 4 and 4 cases of type I–IV, respectively.

Comparison of liver background and accompanying signs between the two groups (Table 3): whether the lower angle of the liver became blunt, peritumoral bile duct dilatation, tumor encasement invading vessels, intrahepatic bile duct dilatation and abdominal lymphadenopathy signs, the difference was statistically significant (*P* < 0.05); liver contour, esophageal/gastric varices, peritumoral bile duct stones, liver capsule shrinkage, vascular tumor thrombus, common bile duct/left and right hepatic duct stones, gallstones/gallbladder absence and abdominal lymph node heterogeneous enhancement and other signs, the difference was not statistically significant (*P* > 0.05). The left lobe of the liver lesions were mainly hepatogastric lymphadenopathy, and the right lobe of the liver lesions were mainly hilar and hepatopancreatic lymphadenopathy.

**Table 3:** Background and accompanying signs of ICC liver in group A and B.

Basic features	Group A (n = 66)	Group B (n = 72)	*P* value
Liver outline			
Smooth	4	2	0.056
Less smooth	48	64	
Serrated	14	6	
Inferior hepatic angle			
Dull	59	50	0.006*
Sharp	7	22	
Esophageal and gastric varices			
Yes	21	14	0.118
None	45	58	
Peritumoral bile duct stones			
Yes	4	7	0.536
None	62	65	
Peritumoral biliary dilatation			
Yes	12	36	0.000*
None	54	36	
Shrinkage of hepatic capsule			
Yes	32	42	0.306
None	34	30	
Vascular tumor thrombus			
Yes	11	12	1.000
None	55	60	
Vessel encasement invasion			
Yes	17	46	0.000*
None	49	26	
Dilated intrahepatic ducts			
Yes	18	33	0.034*
None	48	39	
Choledocholithiasis/left and right hepatic duct			
Yes	2	3	1.000
None	64	69	
Gallbladder stones/absent gallbladder			
Yes	16	9	0.081
None	50	63	
Enlarged lymph nodes			
Yes	45	62	0.014*
None	21	10	
Lymph node heterogeneous enhancement			
Yes	16	14	0.540
None	50	58	

Chi-square test or Fisher 's exact test

*Means *P*value is statistically different.

In addition, for lesions larger than 3 cm in diameter, there were statistically significant differences in peritumoral bile duct dilatation and signs of vascular encasement invasion between groups A and B (*P* = 0.039).

### Comparison of ICC imaging in different pathological differentiation degrees

There were statistically significant differences in signs of vascular encasement invasion between well-to-moderately differentiated groups A and B (*P* = 0.006). Poorly differentiated groups A and B showed statistically significant differences in tumor plain scan density uniformity, signs of tumor encasement invading vessels, and enlarged lymph nodes (*P* = 0.002, 0.001, 0.007) (Table 4).

**Table 4:** Imaging characteristics of ICC lesions in group A and B with different degrees of differentiation.

Basic features	Well-medium differentiated group (n = 27 + 40)		Poorly differentiated group (n = 39 + 32)		*P* value	
	Group A	Group B	Group A	Group B	Well-to-moderately differentiated group	Poorly differentiated group
Morpho-						
Round shape	17	22	27	21	0.551	0.791
Irregular shape	10	18	12	11		
Maximum diameter						
< 3 cm	5	2	4	2	0.149	0.057
3–6 cm	9	14	15	7		
> 6 cm	13	24	20	23		
Boundary						
Clear	10	15	17	9	0.893	0.179
Blurring	17	25	22	23		
Precontrast						
Uniform density	6	6	15	2	0.524	0.002*
Non-uniform density	21	34	24	30		
Arterial phase						
Edge ring high density	15	24	19	18	0.596	0.138
Non-uniform high density	5	3	5	2		
Equal/low density	7	13	15	12		
Overall reinforcement pattern						
Type I	12	20	19	17	0.796	0.088
Type II	7	13	11	13		
Type III	3	3	4	1		
Type IV	5	4	5	1		
Vascular tumor thrombus						
Yes	7	5	6	7	0.512	0.469
None	22	35	33	25		
Vessel encasement invasion						
Yes	7	25	10	21	0.006*	0.001*
None	20	15	29	11		
Enlarged lymph nodes						
Yes	21	33	24	29	0.841	0.007*
None	6	7	15	3		
Lymph node heterogeneous enhancement						
Yes	6	9	10	5	0.894	0.370
None	21	31	29	27		

Chi-square test or Fisher 's exact test

*Represents that *P* values are statistically different.

### Comparison of ICC imaging according to tumor size

There were statistical differences in signs of vascular encasement invasion between small-to-Medium groups A and B (*P* = 0.016). Large groups A and B showed statistical differences in tumor plain scan density uniformity, vascular encasement invasion, arterial phase, overall reinforcement pattern and peritumoral bile duct stones, peritumoral biliary dilatation (*P* = 0.007, 0.001, 0.015, 0.005,0.036,0.000).

### Comparative analysis of pathology and imaging of abdominal lymph nodes

A total of 43 cases of lymph node pathology showed metastasis after operation, including 16 cases in group A and 27 cases in group B (*P* > 0.05). Lymph nodes showed heterogeneous enhancement or homogeneous enhancement on CT enhancement.

### Postoperative follow-up

**Figure 6:**
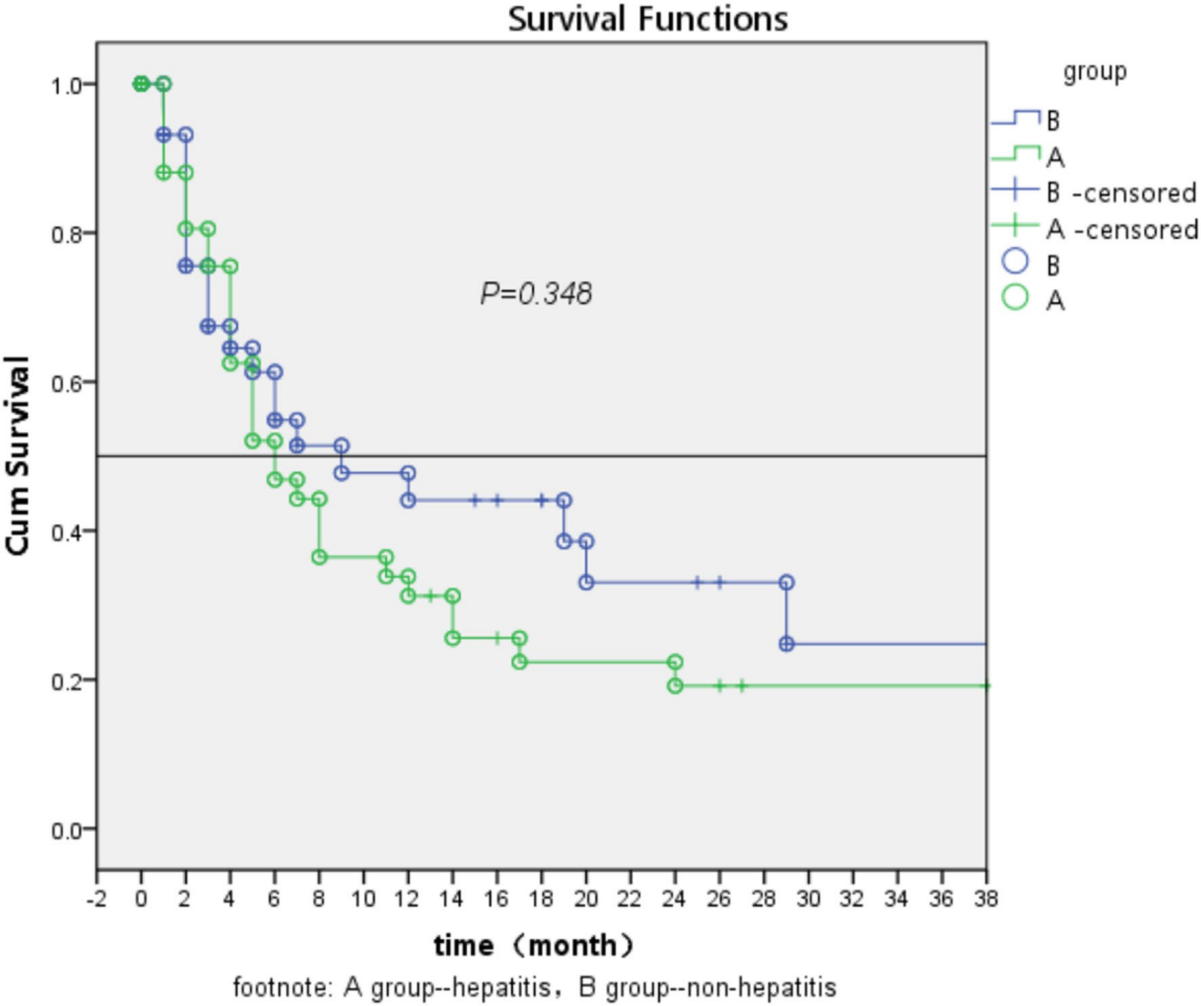
Tumor-free survival rate and follow-up time after ICC in hepatitis and non-hepatitis groups.

Postoperative follow-up ended December 31, 2022. During the follow-up period, there were 78 cases of disease progression, including 44 cases in group A and 34 cases in group B; 43 cases died, including 16 cases in group A and 27 cases in group B; 7 cases had no progression, including 5 cases in group A and 2 cases in group B. There was no significant difference in postoperative progression-free survival time between group A and group B by Log Rank test (*P* > 0.05) (Fig. 6).

## Discussion

The incidence of ICC is increasing, while ICC patients with hepatitis B and cirrhosis show atypical imaging findings, which easily affects the diagnosis of ICC. In recent years, many studies have shown that hepatitis B virus infection is statistically associated with ICC^19,22^. In this study, we investigated the prognostic value of hepatitis virus infection, and we found no significant difference in postoperative tumor progression-free survival between ICC patients with hepatitis and those without hepatitis, however, some clinical, imaging, and pathological characteristics did differ between the two groups.

Our study found that patients in group A had a lower age of predilection and a higher proportion of males, which is similar to other studies^26–28^ , and the early age of onset may be related to the early detection of lesions by regular examination of patients. The positive rate of liver fluke infection in this group of patients was 44.6%, and it has been confirmed in the literature that liver fluke infection is associated with ICC^29^ .

In our study, the positive rate of HBc antibody in the non-hepatitis group was as high as 78.3%. Studies have shown that covalently closed circular DNA^30^ can still be detected in the liver of about 14% of patients after 10 years of HBsAg clearance, while HBc antibody positive represents previous infection with hepatitis B virus. A study showed that IAHBc is an independent prognostic factor for tumor resection in ICC patients after curative recurrence^31^. Is HBc antibody positive alone also involved in the occurrence of intrahepatic cholangiocarcinoma? It can be further investigated in the future.

In terms of tumor markers, AFP was elevated in Group A in a higher proportion, possibly as a result of chronic hepatitis B infection, cirrhosis, or liver cancer^32,33^; AFP could also be elevated when ICC originated from liver stem cells or precursor cells^34^ . The positive rate of CA125 in group B was higher, which was consistent with the results in the 2021 NCCN Guidelines for the Diagnosis and Treatment of Hepatobiliary Carcinoma^33^ ; CA125 is a macromolecular polysaccharide protein, which has good stability in serological levels compared with CA199 and AFP and is not easily interfered by factors of liver, biliary tract inflammation, or cholelithiasis^35^ .

In this two groups, the right lobe of liver was the main site of tumor distribution. In group A, the proportion of lesions located in the peripheral part was higher, and the lesion diameter was smaller and the density was uniform, consistent with that reported in the literature^25^ , presumably because the high rate of reexamination of chronic hepatitis B led to a high rate of early tumor detection. In Group B, the tumor margin was irregular, prone to microvascular invasion, and the prognosis was also poor^36^ .

In terms of liver background, there were statistical differences in the inferior hepatic angle, but there was no significant difference in the liver outline and esophageal and gastric varices, suggesting that most of the group B also had chronic liver disease caused by non-hepatitis B infection, and chronic liver disease can be caused by a variety of etiologies, fatty liver disease has now surpassed chronic hepatitis B infection as the most important cause in China^37^**.** This study did not focus on the study of fatty liver in patients.

Previous studies have found that ICC enhancement in the setting of chronic hepatitis B and cirrhosis is usually more pronounced than that in the setting without chronic liver disease and cirrhosis^38^. This enhancement feature is similar to HCC in the setting of cirrhosis^25^ , so ICC is easily misclassified as LR-5/5v in 8/35 patients in the Liver Imaging Reporting and Data System (LI-RADS)^39^ . Therefore, it is not enough to diagnose ICC associated with chronic hepatitis B by different enhancement characteristics in the arterial phase alone, which should be combined with more signs. In the overall enhancement pattern, there was no statistically significant difference between the two groups, and ICC was mainly dominated by early rim hyperenhancement + internal delayed hypoenhancement (type I) or progressive hypoenhancement (type II) in both groups, but 8 cases in group A showed heterogeneous hyperenhancement followed by decline, which was similar to the typical HCC "clearance" pattern^33^ .

In the accompanying signs, there were 6 cases of peritumoral bile duct stones, 30 cases of peritumoral bile duct dilatation , and 27 cases of overall intrahepatic bile duct dilatation in group A. It was speculated that peritumoral bile duct dilatation was partially caused by bile duct obstruction and partially caused by liver fluke infection. Kim et al. reported that risk factors for ICC associated with hepatolithiasis are older age, distal bile duct dilatation in proximal bile duct strictures, liver atrophy, bile duct stones in the left lobe, and postoperative stone recurrence^3^ ; in addition, liver fluke infection can also cause cholangitis lesions and then lead to malignant transformation of bile duct epithelial cells. In addition, peritumoralvascular encasement and invasion were more common in group B, which is similar to a study of 155 ICC cases^14^. Another meta-analysis showed a comparable proportion of vascular encasement and invasion^40^, which differed from the results of this study, and we speculated that it may be that the proportion of lesions showing large diameters was higher in the non-hepatitis group in this study, when the tumor was large and there was a high possibility of vascular encasement invasion. Another factor could be the small sample size of this study.

In terms of pathological differentiation, there were differences in signs of vascular encasement invasion between group A and group B, The degree of differentiation did not have a significant effect in it. The proportion of lymphadenopathy was higher in poorly differentiated group B, on the one hand because biliary inflammatory lesions stimulated reactive hyperplasia of abdominal lymph nodes more in group B, and on the other hand, the malignancy of ICC may be higher in group B. This may suggest that when imaging finds lymphadenopathy in the hepatitis B virus-negative ICC patients, it represents that the tumor may be poorly differentiated and requires more aggressive adjuvant therapy and a wider extent of lymph node dissection.

Studies have demonstrated that lesion size may influence the imaging appearance of ICC^41^, but there were statistical differences in tumor plain scan density uniformity, signs of vascular encasement invasion, arterial phase, overall reinforcement pattern and peritumoral bile duct stones, peritumoral biliary dilatation in large groups A and B. This may be due to the higher malignancy in group B. Group A showed a greater proportion of edge ring high density in the arterial phase of lesions. Thus, there were more instances of early rim hyperenhancement + internal delayed hypoenhancement in overall reinforcement pattern. This enhancement feature is similar to HCC in the setting of cirrhosis^25^

In recent years, the impact of hepatitis B virus infection on the prognosis of ICC patients remains controversial.Most previous studies have insisted that hepatitis B virus infection may be a favorable prognostic factor in ICC patients^19,40,43,44^**.** However, in this study, there was no statistically significant difference in the survival time without postoperative tumor progression between groups A and B, and the results of this study were also similar to those of some studies^42^. Ahn et al.^42^ analyzed the survival outcomes of 37 HBV-associated and 255 HBV-negative ICC patientswho underwent hepatic resection, and demonstrated that their postoperative favorable outcomes showed no significant difference, but more favorable tumor featureswere observed in HBV-associated ICC patients due to a relatively early diagnosis. So it is expected to increase the sample size and multicenter data in the future to investigate the effect of chronic hepatitis B on the prognosis of ICC. At present, there are no consensus diagnostic procedures and treatments for such populations^45^. We need to carefully consider the establishment of clinical therapeutic strategies for patientswith HBV-associated ICC and clinical trials assessing managements.

There were several limitations in this study. Firstly, we only assessed CT features in multiphase CT, features from MRI and radiomic features from both CT and MRI are worth exploring to further improve accuracy. Second, the differences in gender and age between the two groups were statistically significant, we did not use a propensity match score to compare both groups. In addition, in our study, whether patients participated in the HCC surveillance program was not investigated, time to diagnosis affects the tumor staging and survival.

## Conclusions

Compared with chronic hepatitis B negative ICC patients, chronic hepatitis B positive ICC patients have different clinical, pathological and imaging characteristics, and there is little difference in progression-free survival after surgery. So maybe the same treatment and surveillance policies should be applied regardless of HBV serology. These characteristics may provide help for accurate clinical diagnosis and treatment.

### Supplementary Information

Supplementary Information 1.

Supplementary Information 2.

Supplementary Information 3.

## Data Availability

All data generated or analysed during this study are included in this published article and its supplementary information files.
